# Lightweight Multi-Scale Asymmetric Attention Network for Image Super-Resolution

**DOI:** 10.3390/mi13010054

**Published:** 2021-12-29

**Authors:** Min Zhang, Huibin Wang, Zhen Zhang, Zhe Chen, Jie Shen

**Affiliations:** 1 College of Computer and Information Engineering, Hohai University, Nanjing 211100, China; zhangmin_1233@hhu.edu.cn (M.Z.); zz_hhuc@hhu.edu.cn (Z.Z.); chenzhe@hhu.edu.cn (Z.C.); shenjie_2003045@hhu.edu.cn (J.S.); 2 Department of Information Engineering, Gannan University of Science and Technology, Ganzhou 341000, China

**Keywords:** super-resolution, lightweight, multi-scale, asymmetric multi-weights attention

## Abstract

Recently, with the development of convolutional neural networks, single-image super-resolution (SISR) has achieved better performance. However, the practical application of image super-resolution is limited by a large number of parameters and calculations. In this work, we present a lightweight multi-scale asymmetric attention network (MAAN), which consists of a coarse-grained feature block (CFB), fine-grained feature blocks (FFBs), and a reconstruction block (RB). MAAN adopts multiple paths to facilitate information flow and accomplish a better balance of performance and parameters. Specifically, the FFB applies a multi-scale attention residual block (MARB) to capture richer features by exploiting the pixel-to-pixel correlation feature. The asymmetric multi-weights attention blocks (AMABs) in MARB are designed to obtain the attention maps for improving SISR efficiency and readiness. Extensive experimental results show that our method has comparable performance with fewer parameters than the current advanced lightweight SISR.

## 1. Introduction

Image super-resolution (SR) is the process of recovering a high-resolution (HR) image from a given low-resolution (LR) image. Several corresponding HR images can be generated from a given LR image, which is fundamentally ill-posed. Recently, many researchers have introduced deep learning (DL) to solve the SR problem. In particular, the domain of single-image SR has achieved remarkable performance using deep convolutional neural network (CNN) techniques [1]. Dong et al. [2] built an end-to-end SR convolutional neural network (SRCNN), which obtained significant performance improvement compared to traditional methods. Kim et al. [3] presented a very deep super-resolution (VDSR) network, which increased the depth of the network to 20 layers and reduced training difficulty by residual learning. Lim et al. [4] designed an enhanced deep super-resolution (EDSR) network with an intense architecture with more than 60 layers, acquiring high reconstruction accuracy. To reduce the network depth and extract diversity features, some researchers studied multiple path networks to obtain various features at multiple contextual scales. Liu et al. [5] proposed a residual feature distillation network (RFDN), which learned more discriminative feature representations through multiple feature distillation connections. The SR network design discussed above is of equal importance for all channels and locations. Furthermore, the attention-based network implemented confirms that not all features are essential for SR. Inspired by SENet [6], Zhang et al. [7] employed a residual channel attention network(RCAN) to enhance the results of SR by exploiting the interdependence with the channel attention residual blocks. In addition, the spatial attention mechanism exploited the spatial information of the feature maps for HR image reconstruction. Liu et al. [8] present a residual feature aggregation network (RFANet) using spatial attention to achieve a greater performance improvement, including 30-layer residual feature aggregation.

As mentioned above, although they achieved great success, there were some defects in the structure of SR methods. Firstly, these methods are only suitable for deeper architectural design because of the huge network capacity required. Secondly, multi-path networks mitigate gradient disappearance at the cost of numerous parameters and high computational costs. Finally, the attention mechanism network reduces the parameter overhead at the cost of network capacity. The drawback of the above approaches is that numerous parameters are required to obtain better performance, which is not conducive to real-world applications. Practical applications such as mobile devices (e.g., cell phones) are limited by performance breakthroughs that increase parameters and complexity while imposing high computational costs and huge memory capacity. To address these issues, some researchers have turned their concerns to the construction of lightweight models. The cascading residual network(CARN) [9] utilized the residual cascade network of group convolution to achieve lightweight and efficient reconstruction. The lightweight information multi-distillation network (IMDN) [10] introduced multiple distillation blocks of information to expand the receiving field and then took a step-by-step approach to extract hierarchical features and merge them through channel attention. However, most of the previously proposed lightweight networks have poor model performance with fewer parameters by designing shallow network structures or recursive connections. Meanwhile, these methods ignore the exploration of the correlation of the middle layer features, which makes CNN’s representation ability inadequate [11]. In addition, some advanced lightweight networks introduced attention into deep extraction feature models, which contained channel attention (CA) and spatial attention (SA) [12]. This information tended to recover only high-frequency details of the different functions; CA and SA ignore the relationship between pixels. However, the layer interaction is significant to facilitate the interchange of information. It is essential to find an advanced module to refine the convolutional output within the block so that the whole network can learn more helpful features. 

**Figure 1:**
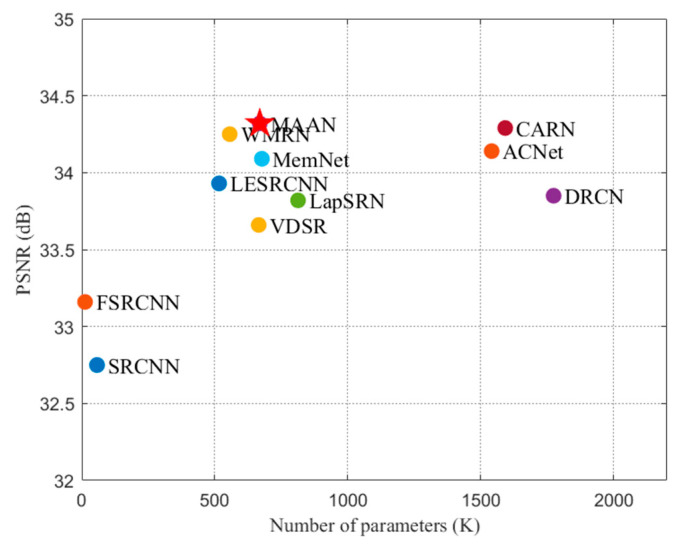
Performance and parameters compared between MAAN (red star) and the existing methods on Set5 with scale factor ×3. MAAN gains the best PSNR with the appropriate parameters.

In this work, we address a lightweight multi-scale asymmetric attention network (MAAN), with a diverse network architecture design to gain better performance. MAAN fully utilizes multiple path aggregation of the middle- and deep-layer features to achieve more accurate extraction. On the one hand, it presents asymmetric multi-weight attention to recover high-frequency feature details and refine important information. On the other hand, a 1 × 1 convolution operation is implemented to reduce the parameters and improve the training efficiency of the network. The peak signal-to-noise ratio (PSNR) is the most commonly used reconstruction quality metric in image SR. The PSNR is determined by the maximum pixel value and the mean square error comparing the reference image to the SR image. The more the parameters, the higher the PSNR value and the quality of the reconstructed image and the better the performance. As Figure 1 shows, compared to the state-of-the-art SR networks, MAAN obtains the best PSNR with appropriate parameters.

Overall, our goal is to propose a lightweight model that optimizes the reconstructed image and achieves the desired trade-off between parameters and computation. The contribution of our work is as follows:

1. We employ fine-grained feature blocks (FFBs) as the backbone module of our framework implementation, which accesses reasonable SR performance with fewer parameters. The multi-scale attention residual block (MARB) of FFBs extracts sufficient multi-scale features for global feature fusion. It enhances asymmetric attention neurons in a larger receptive field to capture richer multi-frequency information features significantly.
2. We propose an asymmetric multi-weights attention block (AMAB) to enhance feature propagation and further extract high-frequency detail features by adaptive selection among the layers.
3. MAAN acquires a better trade-off between performance and lightweight compared to the popular models.

The rest of this paper is structured as follows: Section 2 presents related work on lightweight networks and attention mechanisms in image super-resolution. Section 3 shows the MAAN approach in detail. Section 4 illustrates the experiments and provides important arguments for the proposed technique and shows the experimental performance of SISR. Section 5 concludes the paper.

## 2. Related Work

### 2.1. Lightweight Super-Resolution Networks

To further extend the SR model to mobile device applications, lightweight models have attracted the attention of researchers on how to decrease the number of parameters and computation cost. The deeply recursive convolutional network (DRCN) [13] utilized recursive neural networks to employ a single convolutional layer without including many parameters. The Laplacian pyramid SR network (LapSRN) [14] reconstructed high-resolution images by learning residuals in convolutional layers with step-by-step scaling. To better balance performance and reasoning application, the information distillation network (IDN) [15] effectively combined the characteristics of a global long path and a local short path, which achieved lightweight and efficient reconstruction. Multiple information distillation blocks were introduced into the IMDN [10] to increase the receptive field, which was fused with stratified information through channel attention. The lightweight enhanced SR CNN (LESRCNN) [16] adopted a heterogeneous structure, improving network SR performance by combining low-frequency with high-frequency features. The asymmetric CNN (ACNet) [17] utilized asymmetric convolution to construct hierarchical structure features for adaptively combining local and global information. The multi-scale attention network (MSAN) [18] adopted cascading multiple multi-scale attention blocks and split channel characteristics to further improve performance. Even though the number of lightweight SR methods has grown significantly, it is hard to balance reconstruction accuracy and model capacity. 

In some methods, multi-scale feature extraction via dilated convolution leads to capturing redundant contextual information, while bringing in some non-essential parameters and computational costs. In others, excessive scaling of model parameters makes the image too smooth to better capture the perceptual difference between the model output and the true-value image. Hence, we aim to build a lightweight network, utilizing multiple paths to facilitate information flow and accomplish better information exchange. Accordingly, our study introduces a novel multi-scale block with simple 3 × 3 convolutional combinations to realize the aggregation of different scales and levels of information. Concurrently, channel scaling with asymmetric convolution further reduces parameters and computational costs.

### 2.2. Attention Mechanism

The attention mechanism assigns more priority to specific pixels, which leads to better data processing than others. Recently, the attention mechanism has been widely used in SR to obtain significant features by inhibiting insignificant features. The channel attention mechanism only focused on each channel feature, which computed one-dimensional weights multiplied by channel pixels. Niu et al. [19] presented the holistic attention network (HAN), which fully employs more informative features across layers, channels, and positions for selectively capture. The dense residual Laplacian network (DRLN) [20] proposed a Laplacian pyramidal attention mechanism for learning multiple frequency features. The sparse mask SR (SMSR) [21] explored spatial masks to improve the inference efficiency of SR networks. The SMSR learned to identify “significant” regions in contrast to channel masks. We observe that existing attention modules focus on channel attention or spatial attention, which limits the flexibility of the network to learn 1D and 2D attention weights. SimAM [22] proposed 3D attention weights to refine the feature map in a layer without adding parameters to the original networks. The SimAM module had excellent performance on image classification or object detection.

The attention mechanism still has a lot of room for improvement between accuracy and model capacity. Inspired by SimAM, our study introduces a new attention module AMAB, which identifies significant information by exploring relationships between inter-channel and intra-channel and facilitates the extraction of diverse features, as well as further improving performance with a small number of parameters and computations.

## 3. Methods

### 3.1. Network Architecture

**Figure 2:**
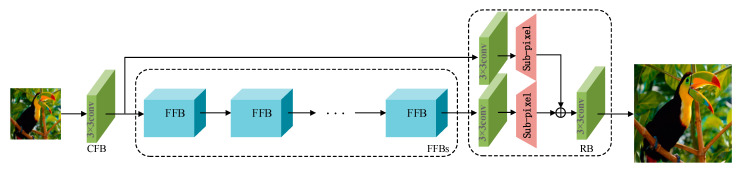
The network framework of the proposed MAAN, which comprises three stages: coarse-grained feature block (CFB), fine-grained feature blocks (FFBs), and reconstruction block (RB). CFB means a 3 × 3 convolution, the core of the structure contains i FFB modules. Lastly, we add an upsampled image to the reconstructed output.

In this section, our lightweight and efficient MAAN is employed. MAAN consists of three main components: coarse-grained feature block (CFB), fine-grained feature blocks (FFBs), and reconstruction block (RB), as depicted in Figure 2. We represent the LR image, the HR image, and the SR image, respectively, as $I_{LR}$, $I_{HR}$, and $I_{SR}$.

Firstly, the input is processed by the CFB. We extract coarse-grained features via only one 3 × 3 convolution layer for lightweight design. The CFB block can be formulated as follows:

(1) $$x_{0}=f_{CFB}(I_{LR})$$

where $f_{CFB}(\cdot )$ denotes the operation of CFB. $x_{0}$ is the coarse-grained features, which is used as input to the fine-grained feature block (FFB) for deep feature extraction.

Secondly, the FFB is the core step for extracting high-frequency features. To fully utilize the image features of the CFB block, we utilize multiple paths to further refine the features and gather various features. The specific progress can be expressed as follows:

(2) $$x_{i}=f_{FFB}(x_{i-1})$$

where $f_{FFB}(\cdot )$ denotes the operation of FFB, where $x_{i-1}$ and $x_{i}$ represent the input and the output respectively of the i-th FFB block.

Finally, in the last stage of the model, we reduce artifacts by using an upsampling operation with sub-pixel convolution, and the enlarged features are mapped to the SR image through a 3 × 3 convolution layer. As shown in Figure 2, $x_{0}$ and $x_{i}$ are transmitted to the reconstruction block, $f_{RB}$, via a global residual connection.

(3) $$I_{SR}=f_{RB}(x_{0},x_{i})$$

Hence, MAAN improves the quality of the final reconstruction with a small cost in parameters. It aggregates features from multiple fields of perception to collect rich contextual information for low-resolution to high-resolution mapping, and it enables a more detailed image to be reconstructed. The super-resolved image, $I_{SR}$, can be expressed by:

(4) $$I_{SR}=f_{RB}(f_{FFB}(f_{CFB}(I_{LR}))=f_{MAAN}(I_{LR})$$

We adopt L1 [23] as the loss function. It can be used to minimize the difference between the predicted SR image and the given HR image to train the MAAN for SR, where θ represents the learning parameter, L represents the loss function. Given a training set ${\left\{I_{LR}^{i},I_{HR}^{i}\right\}}_{i=1}^{M}$, the loss function can be formulated as follows:

(5) $$L(\theta )=\frac{1}{M}\sum_{i=1}^{M}\left|\right|f_{MAAN}(I_{LR}^{i})-I_{HR}^{i}{\left|\right|}_{1}$$

### 3.2. Fine-Grained Feature Block

**Figure 3:**
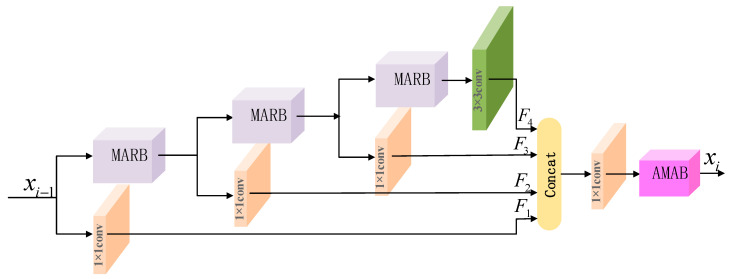
The structure of our proposed the FFB.

As depicted in Figure 3, our FFB is essentially a multiple paths module, which can refine the features in terms of spatial context and produce better information exchange through multiple paths of information flow. FFB is constructed using MARB, AMAB, and 1 × 1 convolutions. FFB utilizes a channel segmentation operation with multiple paths, which divides the input features into two parts. The upper part is retained for MARB operation, and the lower part is compressed into 1 × 1 convolution to extract features. $f_{MARB}(\cdot )$ represents the operation of MARB, each branch is defined as follows:

(6) $$\left\{ \begin{array}{l} F_{1}=C_{1\times 1}(x_{i-1})\\ F_{2}=C_{1\times 1}(f_{MARB}(x_{i-1}))\\ F_{3}=C_{1\times 1}(f_{MARB}(f_{MARB}(x_{i-1})))\\ F_{4}=C_{3\times 3}(f_{MARB}(f_{MARB}(f_{MARB}(x_{i-1})))) \end{array}\right.$$

The concatenated features of multiple branches are fused by a convolution operation with 1 × 1 kernel size. Then, AMAB is applied to significantly enhance the feature flow, allowing higher weights to be assigned to more important features and high-frequency refining details. It can be expressed as

(7) $$x_{i}=f_{AMAB}(C_{1\times 1}[F_{1},F_{2},F_{3},F_{4}])$$

where $\left[F_{1},F_{2},F_{3},F_{4}\right]$ denotes the concatenation of aggregated features. $C_{k\times k}$ denotes the convolution operation with k × k kernel size. $f_{AMAB}(\cdot )$ is defined asymmetric multi-weights attention block.

### 3.3. Multi-Scale Attention Residual Block

When feature extraction is carried out through the convolution kernel with a fixed scale, the ability of network reconstruction is limited by the local feature information. Multi-scale attention residual blocks can enlarge the receptive field and improve computer vision performance. Chen et al. [24] addressed multi-scale feature extraction by dilation convolution and proposed an encoding–decoding image segmentation method, called DeepLabV3+. However, this method directly concatenated features at different scales, which made it difficult to merge this information. To solve the issue, we implemented a new module MARB, which can magnify the receptive field. MARB can employ an attention mechanism to significantly improve the extraction of high-frequency detail features and adopt residual learning to reduce gradient disappearance and facilitate information flow. 

**Figure 4:**
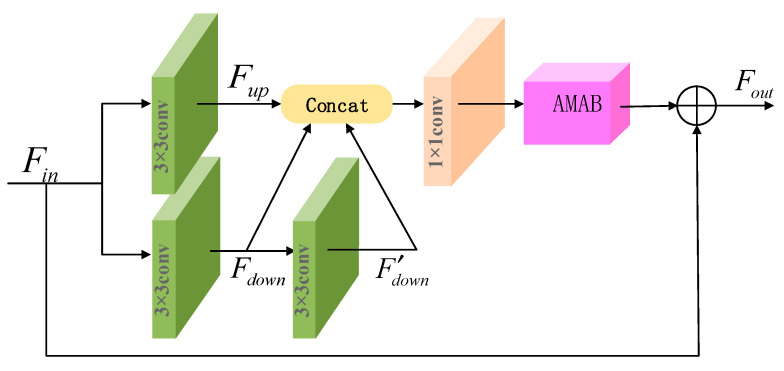
Multi-scale attention residual block structure used in MARB.

As depicted in Figure 4, MARB applies multiple paths to combine the multi-scale features, with one 3 × 3 convolution layer at the top and two 3 × 3 convolution layers at the bottom to expand the perceptual field and achieve better feature correlation. It can be expressed as follows:

(8) $$\left\{ \begin{array}{l} F_{up}=C_{3\times 3}(F_{in})\\ F_{down}=C_{3\times 3}(F_{in})\\ {F^{\prime }}_{down}=C_{3\times 3}(C_{3\times 3}(F_{in})) \end{array}\right.$$

AMAB operation ensures maximum capture of feature information at different scales to achieve better feature relevance. Residual learning for each MARB helps ease the training difficulty of convolution networks and improves the information expression effectively. As mentioned above, this allows MARB to take advantage of available resources to obtain richer information in the SR image. Formally, we describe MARB as follows:

(9) $$F_{out}=F_{in}+f_{AMAB}(C_{1\times 1}[F_{up},F_{down},{F^{\prime }}_{down}])$$

### 3.4. Asymmetric Multi-Weights Attention Block

Each pixel in the image does not exist independently, and they have some correlation with each other. The previous methods always designed channel attention or spatial attention for refining feature maps, thereby ignoring the relation of pixels. Pixel equal treatment is performed either on all channels or on all locations so that the accurate 3D weights can not be computed efficiently. Yang et al. [22] proposed to use 3D attention feature mapping to extract features to compensate for the imperfection of a 1D attention vector or 2D map in extracting features. The linear separability can be used to find the corresponding neurons between a target neuron and other neurons. Borst et al. [25] determined that, for drosophila’s visual orientation selectivity, lobule plate neurons determine the spatial receptive fields of neurons through direction-selective inputs from perceptual neurons T4 and T5 in the fly’s visual system, significantly enhancing preferred directional features and zero-directional features, and performing directional information integration for efficient information flow. Inspired by these, we design an asymmetric multi-weights attention block (AMAB) that can captured the long-range dependencies directly from feature maps. 

Firstly, asymmetric convolutions reinforce the salient features by horizontal and vertical directions, so a k × k convolution is factorized into a k × 1 and a 1 × k kernel [26]. To avoid introducing the computational overhead and extra parameters, the upper branch contains 3 × 1 and 1 × 3 asymmetric convolution kernels. Meanwhile, the 3 × 1 convolution compresses the number of channels with a reduction ratio R, and then another 1 × 3 convolution to expand original channels. We set R = 2, which reduces nearly half of operations and parameters while retaining the same receptive field and optimally balances the number of channels and input/output connectivity. 

**Figure 5:**
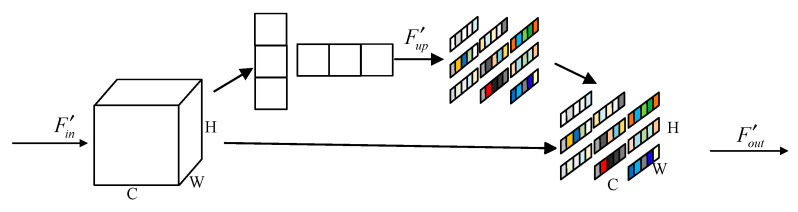
In the structure of asymmetric multi-weights attention block, each pixel in an image has some correlation with other pixels.

As shown in Figure 5, AMAB has three steps: the first step fuses features from horizontal and vertical directions via asymmetric convolutions. It can be calculated as follows:

(10) $${F^{\prime }}_{up}=C_{1\times 3}(C_{3\times 1}({F^{\prime }}_{in}))$$

where ${F^{\prime }}_{up}$ is utilized as the input with multi-weights attention. 

The second step is to extract more effective features using multi-weights attention. All computing is an element-wise operation in the AMAB. Each pixel of the channel and spatial dimensions can be formulated as

(11) $${F^{\prime }}_{out}=\sigma ({F^{\prime }}_{up})\times {F^{\prime }}_{in}$$

where σ(⋅) is the sigmoid function, which does not affect the importance of each pixel, but only the value of the pixel calculation process is limited to avoid excessive overruns. In multi-weights attention, each pixel is interconnected with other pixels, which allows the feature map to more realistically reflect the internal features of the image.

The weight generation is formulated as an energy function to reconstruct the attention mechanism while remaining lightweight. By adaptive selection among various layers, AMAB can capture features of different frequencies. The specific implementation of asymmetric multi-weights attention is shown in Algorithm 1.
**Algorithm 1: The implementation of asymmetric multi-weights attention.**Input X: The feature matrix of H × W × C size.Output X: The resultant matrix of H × W × C size.(1)Set a 3 × 1 convolution layer and compress the channels to C/2.(2)Use a 1 × 3 convolution layer and expand the channels to C.(3)Calculate spatial size N = H × W − 1.(4)Calculate square D = X − X.mean().pow(2).(5)Calculate channel variance through D/N and derive function F for finding the importance of each pixel as F = D/(4 × (v + lambda)) + 0.5, where lambda is the coefficient value.(6)Adding sigmoid to restrict F.(7)Save the value of the output matrix.

## 4. Experiments

### 4.1. Datasets and Metrics

The DIV2K [27] was the source of training and validation data for our model, including the first 800 images as training data and the rest for validation data. We trained the MAAN using the training dataset (DIV2K), which is utilized in most models. We also used four standard benchmark datasets as test datasets, including Set5 [28], Set14 [29], B100 [30], and Urban100 [31]. The original HR training images were downsampled with bicubic interpolation of scale factors ×2, ×3, and ×4, respectively, to obtain the corresponding LR images. The training images were subjected to random rotations of 90°, 180°, and 270° and were manipulated by horizontal flipping. Traditionally, the PSNR has been used for the evaluation of computer vision tasks. However, the perception of structural information within images is measured by structure similarity (SSIM). Then, human vision is more sensitive to changes in luminance. The experiment results are calculated on the PSNR and SSIM by performing on the luminance (Y) channel of the converted YCbCr space. During the training stage, LR images were split into 64 × 64 patches, and the mini-batch size is set to 16. Our network adopted the ADAM optimizer [32] with β1 = 0.9; β2 = 0.999; and ε = 1 ×10^−8^ to minimize the loss function. The initial learning rate was taken as lr = 1 × 10^−4^ and halved for every 25,000 epochs. To ensure that our proposed MAAN had a lower model capacity, we set the number of FFBs to i = 4 and set C = 40 as the number of channels. We constructed our network utilizing Pytorch with an RTX 3080 GPU of 12G memory on the R5-5600 machine.

### 4.2. Model Analysis

#### 4.2.1. Number of FFBs

To better balance model capacity and reconstruction accuracy, we conducted experiments with different numbers of FFBs. As shown in Table 1, we analyzed the number of FFBs with scale factor ×3 on Urban100, the performance of SR can be improved as i grows, accompanying computational cost and parameter increase. To ensure that the proposed model is lightweight enough, we set i = 4 as the final model.

#### 4.2.2. Effect of Reduction Ratio R Setting in AMAB

**Figure 6:**
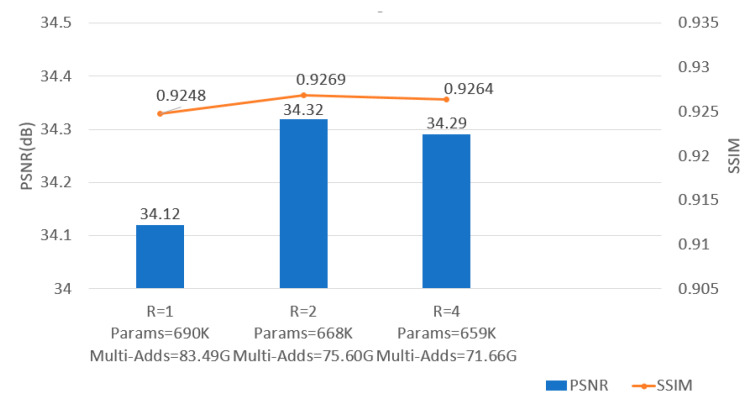
Results of the effect of the asymmetric convolution setting in AMAB with scale factor ×3 on Set5.

For analyzing the value of reduction ratio R in asymmetric convolution, we conducted two extra models for comparison. We set R = 1 and R = 4, respectively. In Figure 6, compared to the first two models, MAAN obtained the best results with the advantages of split channels, making the value of PSNR increase dramatically from 34.12 to 34.32, and the SSIM value consequently improved by 0.0021. Simultaneously, the number of parameters decreased by 28 K, and the computational cost, i.e., multi-ddds, dropped by 7.89G. Asymmetric convolution improved feature representation through channel changes. However, if the number of channels is compressed too low, there will also be a loss of some detailed features. Meanwhile, these changes also imply that effectively using the correlation of asymmetric multi-weights attention within the image can significantly assist in extracting accurate features from the image.

#### 4.2.3. Effect of AMAB

In order to evaluate the superiority of the AMAB, we provided two models for comparison. We first replaced the AMAB with a plain channel attention (CA), namely MAAN-CA. Then, we removed the AMAB to obtain a MAAN-NOAMAB. As shown in Table 2, the performance of the MAAN-NOAMAB was much lower than that of the original MAAN, with a 0.10 dB drop in PSNR value. At the same time, the PSNR and SSIM values of MAAN-CA were 0.05 dB and 0.0007 less than our model, respectively. Notably, our proposed AMAB only had a small increased cost of a few extra parameters and memory with a higher reconstruction accuracy. These results prove the effectiveness and rationality of the AMAB.

### 4.3. Comparison with State-of-the-Art Methods

To verify the advantages of our model, we compare the MAAN with several state-of-the-art SR methods in terms of quantitative and qualitative evaluation, such as SRCNN [2], FSRCNN [33], VDSR [3], DRCN [13], LapSRN [14], MemNet [34], CARN [9], LESRCNN [16], ACNet [17], and WMRN [35].

#### 4.3.1. Quantitative Evaluation

The quantitative evaluation results concerning the average PSNR and SSIM over the four benchmark datasets are shown in Table 3. For a more intuitive comparison, we give the parameters and multi-adds. The parameters of the network model were derived from the number of operations computed in the convolutional window, i.e., generated by the output convolutional elements. In addition, multi-adds was employed to evaluate the model’s computational complexity. It indicates the number of complex product operations for a single image. The multi-adds were computed with a 1280 × 720 output image. Overall, our model with nearly 668K parameters showed better reconstruction accuracy in terms of objective quality scores on most benchmark datasets. Most of the quantitative results of MAAN were either the best or the second-best from a lightweight modeling perspective. For the scale factor ×2, the PSNR gain of MAAN was slightly lower than that of the WMRN by 0.01 dB in Set5 and slightly lower than CARN, which was 0.01 dB in Set14. Unfortunately, CARN suffered from enormous network parameters and computational overhead. For the scale factor ×3, MAAN achieved the best SSIM of all methods and was superior to other modules for the PSNR value except for CARN. For the scale factor × 4, MAAN outperformed most methods and achieved comparable results running very few operations, which takes up fewer multi-adds with more moderate parameters. These advantages indicate that MAAN has a good reconfiguration capability and tends to produce high-quality human perception. Moreover, it can be found that existing models with fewer parameters have lower performance than our model. For example, although the multi-adds value of LESCRNN is much lower than that of our model, it has unsatisfactory results. Compared to the MWRN, our method achieved a performance improvement with slightly more parameters. These results prove the superiority of our proposed MAAN over the advanced models in attaining lightweight and efficient accuracy.

#### 4.3.2. Qualitative Evaluation

**Figure 7:**
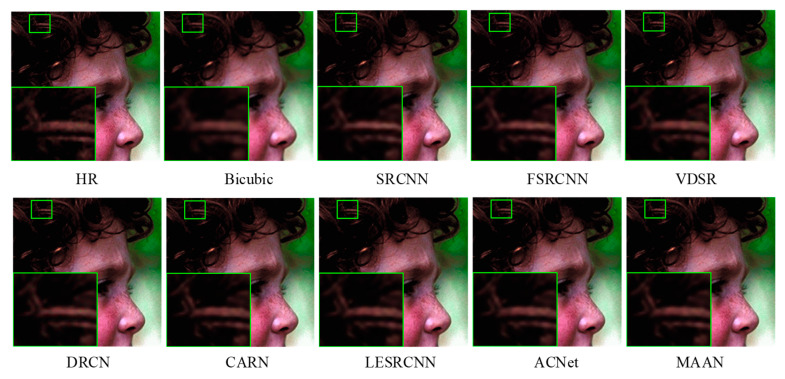
Qualitative comparison over Set14 for scale factor ×2.

**Figure 8:**
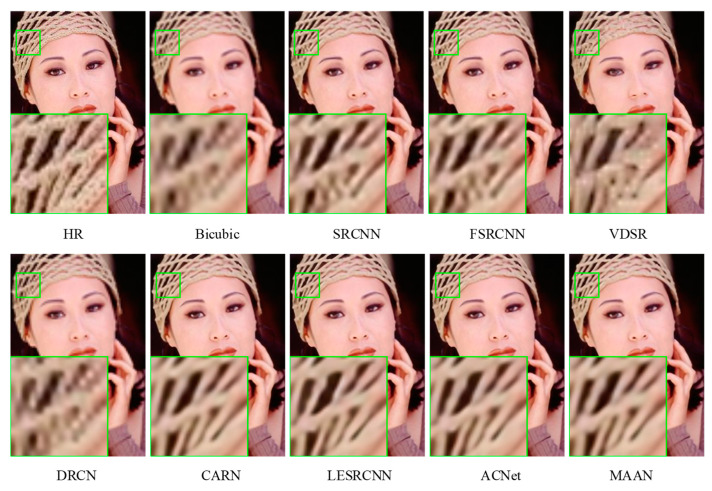
Qualitative comparison over Set5 for scale factor ×3.

**Figure 9:**
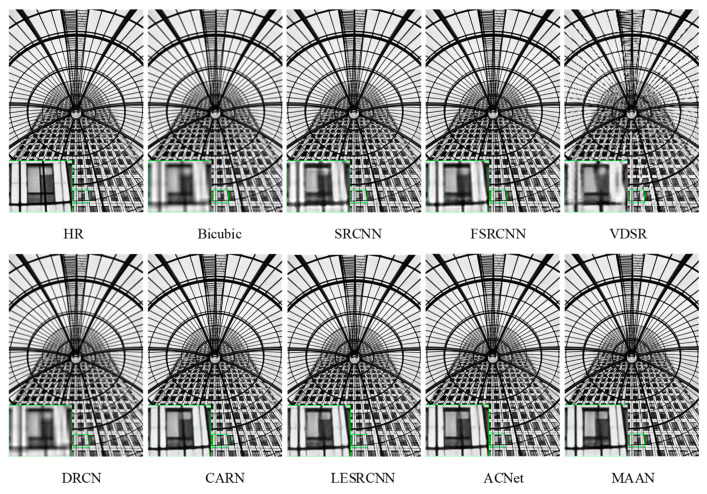
Qualitative comparison over Urban100 for scale factor ×4.

Figure 7, Figure 8 and Figure 9 show a visual comparison of the different scale factors on the benchmark dataset. In Figure 7, MAAN shows qualitative comparison over Set14 for scale factor ×2. Many methods cannot reconstruct the enlarged outline of the left side of the boy’s hair strands, whereas MAAN can recover the hair details well, fully reflecting the role of AMAB and allowing a complete recovery of high-frequency details. In Figure 8, MAAN displays qualitative comparison over Set5 for scale factor ×3, most methods reconstruct images with severe blurring artifacts and fail to restore headpieces clearly. In contrast, MAAN removes artifacts and recovers a higher-quality image. Qualitative comparison over Urban100 for scale factor ×4 was as depicted in Figure 9, although CARN, LESRCNN, and ACNet can produce slightly sharper lines, their lines suffer from significant distortions. In comparison, MAAN combines multi-scale features to expand the receptive fields to capture richer multi-frequency information features. MAAN can overcome this point and have the effect of more accurately reflecting the details of the HR image, thus reconstructing satisfying results.

## 5. Conclusions

In this paper, we present a lightweight MAAN for solving image SR tasks. MAAN first extracts low-resolution features by CFB. Then, the FFB utilizes multiple paths to complement the information exchange. Meanwhile, MARB can extend the perceptual field by extracting feature information at different scales. To further extract high-frequency detail features, an attention mechanism was introduced. AMAB in MARB assigns higher weights to more important features to learn all the previous layers better. Finally, the reconstruction module employed a combination of low- and high-frequency features to capture SR features more robustly. Experiments show that our final model, the MAAN, can achieve comparable performance to state-of-the-art lightweight models.

In the future, we will apply AMAB to improve the performance of water surface video super-resolution that requires more efficiency and lighter weight. MAAN is more suitable for small networks to be applied to other image tasks.

## Figures and Tables

**Table 1:** Analysis of the number of FFB with scale factor ×3 on Urban100.

The Number of FFB	Params	Multi-Adds	PSNR/SSIM
i = 2	342K	39.39G	27.66/0.8422
i = 4	668K	75.60G	28.02/0.8498
i = 6	993K	111.82G	28.20/0.8535

**Table 2:** Ablation study of AMAB with scale factor ×3 on Set14.

Model	CA	N-AMAB	AMAB	Params	Multi-Adds	PSNR/SSIM
MAAN-CA	✓			668K	75.60G	30.25/0.8401
MAAN-NOAMAB		✓		639K	68.52G	30.17/0.8393
MAAN			✓	668K	75.60G	30.27/0.8408

**Table 3:** Quantitative comparison over state-of-the-art SR methods on PSNR/SSIM. MAAN is our method. The red/blue text depicts the best results and the second best ones, respectively.

Scale	Model	Params	Multi-Adds	Set5	Set14	B100	Urban100
×2	SRCNN	57K	52.7G	36.66/0.9524	32.42/0.9063	31.36/0.8879	29.50/0.8946
	FSRCNN	12K	6.6G	37.00/0.9558	32.63/0.9088	31.53/0.8920	29.88/0.9020
	VDSR	665K	612.6G	37.53/0.9587	33.03/0.9124	31.90/0.8960	30.76/0.9140
	DRCN	1774K	17974G	37.63/0.9588	33.04/0.9118	31.85/0.8942	30.75/0.9133
	LapSRN	813K	29.9G	37.52/0.9590	33.08/0.9130	31.80/0.8950	30.41/0.9100
	MemNet	677K	2662.4G	37.78/0.9597	33.28/0.9142	32.08/0.8978	31.31/0.9195
	CARN	1592K	222.8G	37.76/0.9590	33.52/0.9166	32.09/0.8978	31.33/0.9200
	LESRCNN	516K	110.6G	37.65/0.9586	33.32/0.9148	31.95/0.8964	31.45/0.9206
	ACNet	1356K	501.5G	37.72/0.9588	33.41/0.9160	32.06/0.8978	31.79/0.9245
	WMRN	452K	103G	37.93/0.9603	33.49/0.9169	32.13/0.8991	31.83/0.9253
	MAAN	596K	170G	37.92/0.9604	33.51/0.9174	32.14/0.8997	31.86/0.9259
×3	SRCNN	57K	52.7G	32.75/0.9090	29.28/0.8209	28.41/0.7863	26.24/0.7989
	FSRCNN	12K	5.0G	33.16/0.9140	29.43/0.8242	28.53/0.7910	26.43/0.8080
	VDSR	665K	612.6G	33.66/0.9213	29.77/0.8314	28.82/0.7976	27.14/0.8279
	DRCN	1774K	17974G	33.85/0.9215	29.89/0.8317	28.81/0.7954	27.16/0.8311
	LapSRN	813K	149.4G	33.82/0.9227	29.87/0.8320	28.82/0.7980	27.07/0.8280
	MemNet	677K	2662.4G	34.09/0.9248	30.00/0.8350	28.96/0.8001	27.56/0.8376
	CARN	1592K	118.8G	34.29/0.9255	30.29/0.8407	29.06/0.8034	28.06/0.8493
	LESRCNN	516K	49.1G	33.93/0.9231	30.12/0.8380	28.91/0.8005	27.70/0.84152
	ACNet	1541K	369G	34.14/0.9247	30.19/0.8398	28.98/0.8023	27.97/0.8482
	WMRN	556K	57G	34.25/0.9263	30.26/0.8401	29.04/0.8033	27.95/0.8472
	MAAN	668K	75.6G	34.32/0.9269	30.27/0.8408	29.05/0.8042	28.02/0.8498
×4	SRCNN	57K	52.7G	30.48/0.8628	27.49/0.7503	26.90/0.7101	24.52/0.7221
	FSRCNN	12K	4.6G	30.71/0.8657	27.59/0.7535	26.98/0.7150	24.62/0.7280
	VDSR	665K	612.6G	31.35/0.8838	28.01/0.7674	27.29/0.7251	25.18/0.7524
	DRCN	1774K	17974G	31.53/0.8854	28.02/0.7670	27.23/0.7233	25.14/0.7510
	LapSRN	813K	149.4G	31.54/0.8850	28.19/0.7720	27.32/0.7280	25.21/0.7560
	MemNet	677K	2662.4G	31.53/0.8854	28.02/0.7670	27.23/0.7233	25.14/0.7510
	CARN	1592K	90.9G	32.13/0.8937	28.60/0.7806	27.58/0.7349	26.07/0.7837
	LESRCNN	516K	28.6G	31.88/0.8903	28.44/0.7772	27.45/0.7313	25.77/0.7732
	ACNet	1784K	347.9G	31.83/0.8903	28.46/0.7788	27.48/0.7326	25.93/0.7798
	WMRN	536K	45.7G	32.14/0.8944	28.58/0.7804	27.54/0.7342	26.00/0.7816
	MAAN	653K	42.6G	32.21/0.8947	28.58/0.7811	27.55/0.7355	26.01/0.7840

## Data Availability

The datasets generated during and/or analyzed during the current study are available from the corresponding author on reasonable request.
